# Nomogram to Predict the Survival of Chinese Patients with Alcohol-Related Liver Disease

**DOI:** 10.1155/2021/4073503

**Published:** 2021-09-27

**Authors:** Fangfang Duan, Chen Liu, Yuwei Liu, Chunyan Chang, Hang Zhai, Huichun Xing, Jun Cheng, Song Yang

**Affiliations:** ^1^Division 3, Department of Hepatology, Beijing Ditan Hospital, Capital Medical University, Beijing 100015, China; ^2^Division 2, Department of Hepatology, The Fourth People's Hospital of Qinghai Province, Xining, 810000, China

## Abstract

**Objectives:**

Alcohol-related liver disease is an increasing public health burden in China, but there is a lack of models to predict its prognosis. This study established a nomogram for predicting the survival of Chinese patients with alcohol-related liver disease (ALD).

**Methods:**

Hospitalized alcohol-related liver disease patients were retrospectively enrolled from 2015 to 2018 and followed up for 24 months to evaluate survival profiles. A total of 379 patients were divided into a training cohort (*n* = 265) and validation cohort (*n* = 114). Cox proportional hazard survival analysis identified survival factors of the patients in the training cohort. A nomogram was built and internally validated.

**Results:**

The 3-month, 6-month, 12-month, and 24-month survival rates for the training cohort were 82.6%, 81.1%, 74.3%, and 64.5%, respectively. The Cox analysis showed relapse (*P*=0.001), cirrhosis (*P*=0.044), liver cancer (*P* < 0.001), and a model for end-stage liver diseases score of ≥21 (*P*=0.041) as independent prognostic factors. A nomogram was built, which predicted the survival of patients in the training cohort with a concordance index of 0.749 and in the internal validation cohort with a concordance index of 0.756.

**Conclusion:**

The long-term survival of Chinese alcohol-related liver disease patients was poor with a 24-month survival rate of 64.5%. Relapse, cirrhosis, liver cancer, and a model for end-stage liver disease score of ≥21 were independent risk factors for those patients. A nomogram was developed and internally validated for predicting the probability of their survival at different time points.

## 1. Introduction

According to a report from the World Health Organization in 2018, alcohol consumption causes 3.3 million deaths every year, accounting for 5.3% of the global death rate [1]. Excessive drinking has become a significant public health problem. The Global Health Estimates database shows that alcohol-related liver disease (ALD) patients account for 20% of the patients that die of cirrhosis and other chronic liver diseases and 35.5% of the patients that die of liver cancer in mainland China [2]. Our study showed that, in Beijing City, the prevalence of ALD was 4.1% in adult patients [3], and previous research shows that the ratio of ALD in hospitalized liver disease patients is increasing in mainland China [4].

The prognosis of ALD patients has high heterogeneity. Studies show that age [5, 6], continuous drinking [6, 7], smoking [6, 7], and similar lifestyle characteristics are related to the prognosis of ALD patients. Predictors for prognosis of patients with ALD used in research include age-bilirubin-International Normalized Ratio-creatinine score [8], Maddery discriminant function [9], Child–Pugh score [10], and model for end-stage liver diseases (MELD) score [11], especially in alcoholic hepatitis (AH). Yet, research on the prognosis of Chinese patients with ALD is limited. In particular, nomograms for predicting the prognosis of Chinese patients with ALD is seldomly reported. In this study, we built and validated a nomogram to predict the prognosis of Chinese patients with ALD.

## 2. Methods

### 2.1. Patients and Study Design

**Figure 1:**
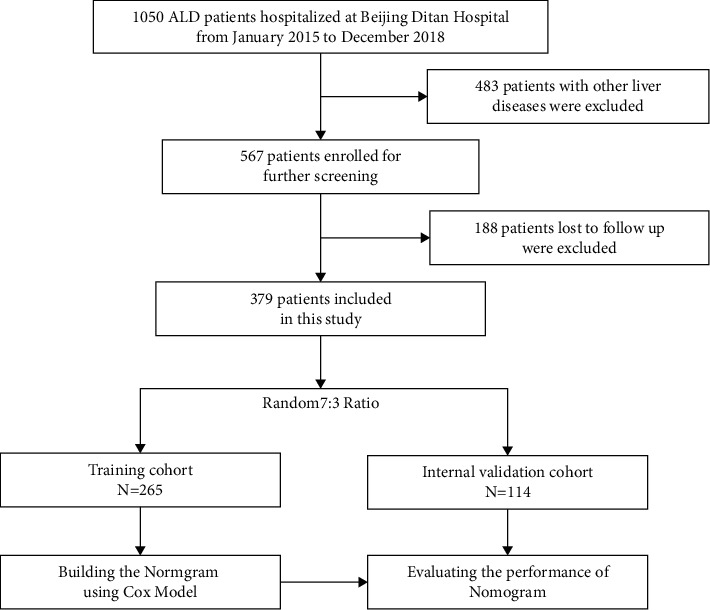
Flowchart of the study's design.

Consecutive adult ALD patients hospitalized in Beijing at Ditan Hospital of Capital Medical University were retrospectively enrolled from January 2015 to December 2018. Diagnosis of ALD and AH was made according to EASL Clinical Practice Guidelines: Management of alcohol-related liver disease [12]. Diagnosis of primary hepatic cancer was made according to Hepatobiliary Cancers, Version 2.2021, NCCN Clinical Practice Guidelines in Oncology [13]. The exclusion criteria were as follows: (1) patients comorbid with viral hepatitis, drug-induced liver injury, autoimmune liver disease, and other liver diseases; (2) patients with previous liver transplantation; and (3) patients missing follow-up. A total of 1050 hospitalized patients with ALD were screened from the hospital information system. A total of 483 patients were excluded for comorbidity with other liver diseases, and 188 patients who were lost to follow-up were excluded. Finally, 379 patients were included in the analysis. The patients were randomly divided into a training cohort including 265 (70%) patients and a validation group including 114 (30%) patients (Figure 1). This study was approved by the Ethics Committee of Beijing Ditan Hospital of Capital Medical University. Informed Consent was waived.

Baseline demographic characteristics, including gender, age, smoking history, and recidivism; laboratory test results including complete blood count, coagulation test, liver function, and renal function; occurrence of decompensation complications of liver cirrhosis such as ascites, hepatic encephalopathy, and oesophageal-gastric varices bleeding; and liver cancer data were collected.

### 2.2. Follow-Up

Patients were followed up with every 3 to 6 months by electronic medical records or telephone interview. We followed up the survival and alcohol withdrawal of each patients within 2 years of hospitalization. The follow-up time was 24 months, and the main end point was death or liver transplantation.

### 2.3. Statistical Analysis

Continuous variables were expressed as mean (±standard) or median (interquartile range (IQR)) and were compared using the independent sample *t*-test or the Mann–Whitney *U*-test. Categorical variables were expressed as count and percentages and were compared using the chi-squared test or Fisher's exact test. A survival curve was constructed using the Kaplan–Meier method and compared with the log-rank test. Univariate analysis was first performed, and the variables that were identified as significant were entered into the multivariate Cox proportional hazard regression analysis used to identify independent prognostic factors. A nomogram was constructed to predict the survival rate of the ALD patients based on the results of the multivariate analysis. The performance of the model was internally evaluated by the concordance index (C-index), which was internally measured by comparing the nomogram-predicted probability with the observed probability. Statistical analysis was performed using SPSS 19.0 and R software (R Foundation for Statistical Computing, Vienna, Austria).

## 3. Results

### 3.1. Patient Characteristics

Baseline characteristics of 379 patients (265 in the training cohort and 114 in the validation cohort) are summarized in Table 1. The baseline demographic indicators, laboratory parameters, decompensation complications of liver cirrhosis and liver cancer, and MELD score were comparable between the two cohorts (all *P* values >0.05).

### 3.2. Survival

**Figure 2:**
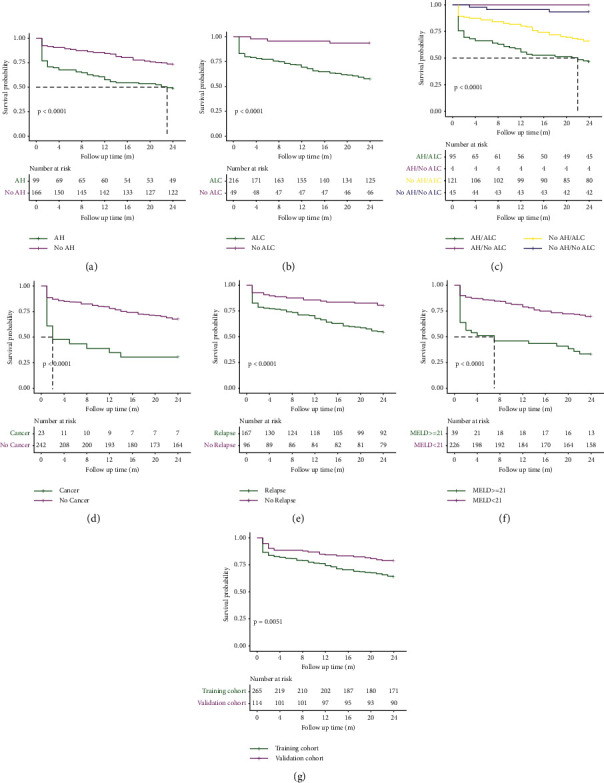
Kaplan–Meier estimate of the ALD patients stratified by cohort. AH/No ALC: patients with alcoholic hepatitis and without cirrhosis due to alcohol-related liver disease; No AH/No ALC: patients with neither alcoholic hepatitis nor cirrhosis due to alcohol-related liver disease; No AH/ALC: patients with cirrhosis due to alcohol-related liver disease and without alcoholic hepatitis; and AH/ALC: patients with both alcoholic hepatitis and cirrhosis due to alcohol-related liver disease. Kaplan–Meier curves for survival of patients with AH and without AH in the training cohort (a); Kaplan–Meier curves for survival of patients with ALC and without ALC in the training cohort (b); patients were divided into four groups, namely, AH/No ALC, No AH/No ALC, No AH/ALC, and AH/ALC. Kaplan–Meier curves for survival of the patients in the training cohort (c); Kaplan–Meier curves for survival of the patients with liver cancer and without liver cancer in the training cohort (d); Kaplan–Meier curves for survival of patients who followed abstinence and relapsed from abstinence in the training cohort (e); Kaplan–Meier curves for survival of the patients with MELD score ≥21 and with MELD <21 in the training cohort (f); Kaplan–Meier curves for survival of the patients in the training cohort and validation cohort (g).

All patients were followed up for 24 months or until an endpoint event took place. In the training cohort, 94 (35.5%) patients died during the follow-up, and the cumulative survival rate of patients at 3 months, 6 months, 12 months, and 24 months was 82.6%, 81.1%,74.3%, and 64.5%, respectively. Survival of patients in training cohort was further analysed according to different subgroups, as shown in Figure 2. The 24-month survival of patients diagnosed as AH was worse than that of patients without AH (Figure 2(a), *P* < 0.001). The 24-month survival of patients diagnosed with cirrhosis due to alcohol-related disease (ALD cirrhosis) was worse than that of patients without ALD cirrhosis (Figure 2(b), *P* < 0.001). Additionally, we divided the patients into four groups, namely, AH/No ALC, No AH/No ALC, No AH/ALC, and AH/ALC, and compared the survival of these groups. We concluded that the AH/ALC group had the worst survival rate (Figure 2(c), *P* < 0.001).

The 24-month survival of patients diagnosed as liver cancer was worse than that of patients without liver cancer (Figure 2(d), *P* < 0.001). In particular, survival of patients who relapsed from abstinence was worse than that of patients who maintained abstinence (Figure 2(e), *P* < 0.001). The MELD score is widely used for predicting prognosis of patients with end-stage liver diseases. We compared the prognosis of patients with baseline MELD scores of ≥21 and <21. Survival of patients with baseline MELD score ≥21 was worse than that of patients with MELD score <21 (Figure 2(f), *P* < 0.001).

In the validation group, 24 (21.1%) patients died during the follow-up and the cumulative survival rate of patients at 3 months, 6 months, 12 months, and 24 months was 88.6%, 88.6%, 84.2%, and 78.9%, respectively. The overall survival was compared to different groups in the training cohort and validation cohort (Figure 2(g)). Results showed that there was no significant difference in overall survival between these groups.

### 3.3. Independent Prognostic Factors in the Training Cohort

A univariate Cox analysis was first performed for the training group patient data, which identified the following as the most significantly related factors to ALD: relapse, cirrhosis, liver cancer, ascites, encephalopathy, haemoglobin (HB), platelet count (PLT), total bilirubin (TBIL), albumin (ALB), creatine (Cr), prothrombin time-International Standardization Ratio (PT-INR), Na, and MELD ≥21 (Table 2). These variables were entered into the multivariate analysis model, except for PT-INR and Cr, since the three variables had already been included in the MELD score. The results revealed that relapse (*P*=0.001), cirrhosis (*P*=0.044), liver cancer (*P* < 0.001), and a MELD score of ≥21 (*P*=0.041) were independent factors for the prognosis of ALD patients (Table 3).

### 3.4. Establishment and Validation of Nomogram

**Figure 3:**
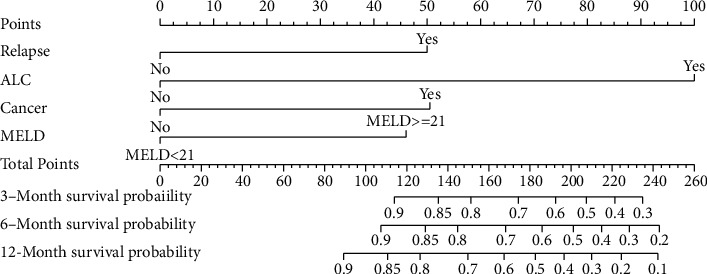
A nomogram for predicting 3-month, 6-month, and 12-month survival of ALD patients. The nomogram was established based on the training cohort.

A nomogram was constructed based on the independent risk factors discovered in the Cox analysis to predict 3-month, 6-month, and 12-month survival rates in the training cohort by weighting the score of each variable. The larger points in the nomogram indicated poorer survival. The nomogram assigned the survival rate by summing the scores identified on the point of scale for each variable. The total prognostic score at the bottom of the scales indicated the probability for 3 months, 6 months, and 12 months (Figure 3).

**Figure 4:**
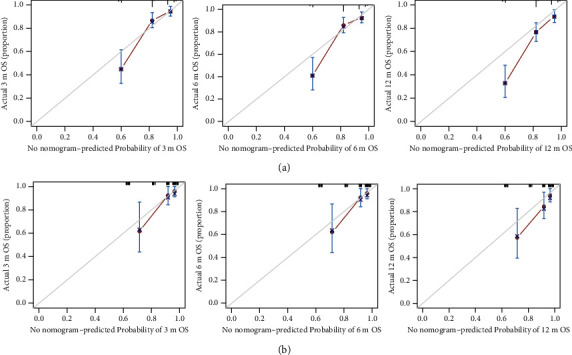
Calibration curve of the nomogram predicting 3-month, 6-month, and 12-month survival rates in the training cohort (a). Calibration curve of the nomogram predicting 3-month, 6-month, and 12-month survival rates in the validation cohort (b).

The nomogram was internally verified through identification and calibration methods, and the calculated C-index was 0.749 (95% confidence interval (CI), 0.702–0.796) in the training cohort. For internal validation, the C-index was 0.756 (95%CI, 0.660–0.852). Lastly, the calibration plots showed good probability consistencies between the nomogram predictions and actual observation (Figure 4).

## 4. Discussion

In this study, we found overall 24-month survival rate of Chinese ALD patients to be 64.5%. Relapse from abstinence, cirrhosis, liver cancer, and a MELD score of ≥21 were the independent prognostic factors of ALD patients. We then built and validated a visible nomogram model to predict the prognosis of ALD patients. This was the first nomogram for predicting prognosis of Chinese ALD patients.

We found that relapse was one of the independent factors affecting the prognosis of ALD patients. The 24-month survival of ALD patients was 44.9% among those who were abstinent and 19.4% for these who relapsed to alcohol consumption, which was consistent with other studies [7, 14–16]. One previous study showed that abstinence for 3 months can improve the prognosis of ALD and the histological characteristics of ALD patients at all stages [17]. It is of great importance to identify patients with high-risk rehydration during the follow-up period for early intervention of relapse in patients with ALD. Abstinence promotion should include the multidisciplinary intervention of addiction experts and measures for patient rehydration. This should involve cognitive behaviour therapy based on psychotherapy, motivational enhancement therapy, and comprehensive medical care. [18–21] It was pointed out that baclofen is expected to prevent ALD patients from relapse from abstinence [22]. However, the evaluation of psychotherapy for maintaining abstinence and the effectiveness of drug treatment is still lacking, and further research is needed.

In this study, we also found that cirrhosis and liver-related cancer were independent prognostic factors. Prior studies implied that patients with ALD, especially those with liver cirrhosis, have a significantly increased risk of liver cancer as the disease progresses [23, 24]. In this study, 95.7% of ALD patients with liver cancer also had liver cirrhosis. It has been previously well established that patients with ALD cirrhosis and alcohol-related liver cancer are more likely to progress than patients with liver cirrhosis and liver cancer due to other reasons [25, 26]. In this study, the 24-month mortality of ALD patients with liver cancer was as high as 69.6%. Late diagnosis and poor response to sorafenib and other TKI inhibitors might contribute to the poor survival rate. For patients with alcohol-related HCC, another concern is that alcohol use was related to sustained expression of proto-onco B-Raf (BRAF), which might result in resistance to sorafenib and other TKIs therapy [27]. We found that cirrhosis was the strongest predictor of death in patients with ALD, followed by liver cancer. Therefore, early diagnosis of liver cancer in patients with ALD, especially in patients with liver cirrhosis, is one key factor for the management of ALD patients. As required by some healthcare guidelines, patients with liver cirrhosis should be regularly monitored by ultrasound [28].

It is worth noting that previous studies paid more attention to the survival of patients with ALD cirrhosis and AH, respectively [15, 29–31]. However, there were relatively few studies on patients with AH combined with ALD cirrhosis. In our study, although AH was not an independent prognostic factor for patients with alcoholic liver disease, the K–M curve suggested that the mortality of patients with ALD cirrhosis combined with AH was significantly lower than that of ALD cirrhosis patients without alcoholic hepatitis. This suggests that we should pay more attention to patients with AH in the background of cirrhosis.

MELD score was originally used to predict the survival of patients undergoing selective transjugular intrahepatic portosystemic shunt to prevent oesophageal variceal bleeding or refractory ascites [32]. After that, MELD score was extended to assess the mortality of end-stage liver disease and assist liver transplantation centres in organ allocation [11]. In previous studies, MELD score was shown to have a good predictive value for the survival of patients with AH [33–35]. In our study, we used an MELD score of ≥21 as the cutoff point and applied it to the overall ALD population. We concluded that an MELD score of ≥21 was an independent prognostic factor of ALD patients. In addition, we excluded the MELD score in our multivariate analysis model and added TBIL and Cr to the model. The results showed that Cr was an independent prognostic factor for ALD patients. Considering that Cr was included in the MELD score, the important predictive role of MELD score in the survival of Chinese ALD population was further confirmed.

At the time of this study, there are many predictive models for surgical treatment, radiotherapy, and chemotherapy of ALD [36, 37], but there is a lack of predictive models for medical treatment of ALD patients. This study included most of the baseline indicators that were found to be meaningful in previous studies and constructed a nomogram using the variables of relapse, cirrhosis, cancer, and an MELD score of ≥21. In addition, the model was validated and found to have good sensitivity and accuracy.

As a single-centre, retrospective study, the external validation cohort is unavailable for this study. A multiple-centred, prospective study with a larger sample size is ongoing. Despite this, we successfully built the nomogram with our cohort, and internal validation showed good performance.

In conclusion, the study showed that the 24-month survival rate of Chinese hospitalized ALD patients was only 64.5%, which calls for strengthened management for patients like these. A nomogram with good performance was built to predict the survival in ALD patients, which may have implications for the management of ALD patients.

## Figures and Tables

**Table 1:** Baseline characteristics of patients in different cohorts.

Variable	Training cohort (*n* = 265)	Validation cohort (*n* = 114)	*P* value
Clinical characteristic			
Age, years	55.36 ± 10.83	54.03 ± 12.16	0.290
Male, *n*%	260 (98.1%)	112 (98.2%)	1.000
Relapse, *n*%	167 (63.0%)	65 (57.0%)	0.272
Smoking, *n*%	196 (74.0%)	76 (66.7%)	0.148
Cirrhosis, *n*%	216 (81.5%)	91 (79.8%)	0.701
AH, *n*%	99 (37.4%)	33 (28.9%)	0.115
Cancer, *n*%	23 (8.7%)	10 (8.8%)	0.977
Ascites, *n*%	177 (66.8%)	69 (60.5%)	0.241
Varices bleeding, *n*%	31 (11.7%)	15 (13.2%)	0.690
Encephalopathy, *n*%	38 (14.3%)	15 (13.2%)	0.761

Laboratory parameters			
WBC (10^9^/L)	5.33 (3.93–7.78)	5.55 (3.99–8.45)	0.435
HB (g/L)	113.00 (89.00–133.00)	110.50 (88.58–131.20)	0.796
PLT (g/L)	96.00 (63.70–144.00)	107.20 (58.75–192.25)	0.194
ALT (U/T)	29.30 (18.60–51.35)	27.00 (16.55–52.55)	0.663
AST (U/T)	59.30 (34.60–102.35)	49.35 (36.00–84.53)	0.224
TBIL (*μ*mol/L)	44.00 (20.95–108.15)	33.80 (17.90–75.83)	0.109
ALB (g/L)	30.90 (26.25–36.00)	31.95 (26.58–36.90)	0.418
Cr (*μ*mol/L)	66.90 (56.30–81.70)	63.80 (54.00–79.00)	0.095
PT-INR	1.32 (1.14–1.70)	1.31 (1.08–1.63)	0.236
Na (mmol/L)	138.30 (135.30–141.25)	139.50 (136.15–141.95)	0.083

Scoring system			
MELD score ≥21, *n*%	39 (14.7%)	11 (9.6%)	0.181

Training cohort vs. validation cohort after random allocation with ratio 7 : 3. Data were reported as counts and percentages, median±standard deviation, or medians with 25th and 75th percentile, respectively. AH, alcoholic hepatitis; WBC, white blood cell; HB, haemoglobin; PLT, platelet count; ALT, alanine transaminase; AST, aspartate transaminase; TBIL, total bilirubin; ALB, albumin; Cr, creatine; PT-INR, prothrombin time-International Standardization Ratio; MELD score, model for end-stage liver diseases score.

**Table 2:** Univariable analysis of ALD patients in the training cohort.

Variable	HR	95% CI	*P* value
Clinical characteristics			
Age, years	0.986	0.968–1.005	0.147
Male, *n*%	2.183	0.312–15.259	0.431
Relapse, *n*%	2.781	1.682–4.601	<0.001^*∗*^
Smoking, *n*%	1.085	0.683–1.724	0.728
Cirrhosis, *n*%	8.791	2.785–27.748	<0.001^*∗*^
AH, *n*%	2.461	1.643–3.687	<0.001^*∗*^
Cancer, *n*%	3.462	2.016–5.946	<0.001^*∗*^
Ascites, *n*%	3.011	1.759–5.156	<0.001^*∗*^
Varices bleeding, *n*%	1.166	0.636–2.136	0.619
Encephalopathy, *n*%	1.917	1.170–3.141	0.010^*∗*^

Laboratory parameters			
WBC (10^9^/L)	1.020	0.987–1.053	0.245
HB (g/L)	0.993	0.986–1.000	0.036^*∗*^
PLT (g/L)	0.996	0.993–0.999	0.014^*∗*^
ALT (U/T)	0.999	0.998–1.000	0.204
AST (U/T)	1.000	1.000–1.001	0.061
TBIL (*μ*mol/L)	1.004	1.003–1.006	<0.001^*∗*^
ALB (g/L)	0.908	0.876–0.940	<0.001^*∗*^
Cr (*μ*mol/L)	1.007	1.005–1.009	<0.001^*∗*^
PT-INR	2.098	1.580–2.788	<0.001^*∗*^
Na (mmol/L)	0.985	0.977–0.992	<0.001^*∗*^

Scoring system			
MELD ≥21, *n*%	3.235	2.056–5.090	<0.001^*∗*^

^*∗*^*P* value <0.05 was considered significant. CI, confidence interval; AH, alcoholic hepatitis; WBC, white blood cell; HB, haemoglobin; PLT, platelet count; ALT, alanine transaminase; AST, aspartate transaminase; TBIL, total bilirubin; ALB, albumin; Cr, creatine; PT-INR, prothrombin time-International Standardization Ratio; MELD score, model for end-stage liver diseases score.

**Table 3:** Multivariable analysis of ALD patients in the training cohort.

Variable	HR	95% CI	*P* value
Relapse, *n*%	2.460	1.431–4.227	0.001^*∗*^
Cirrhosis, *n*%	3.875	1.036–14.500	0.044^*∗*^
AH, *n*%	0.937	0.572–1.534	0.796
Cancer, *n*%	2.888	1.625–5.132	<0.001^*∗*^
Ascites, *n*%	1.365	0.726–2.566	0.334
Encephalopathy, *n*%	1.458	0.879–2.419	0.144
HB	1.000	0.992–1.008	0.937
PLT	0.998	0.995–1.001	0.231
ALB	0.963	0.919–1.009	0.115
PT-INR	1.018	0.602–1.721	0.947
Na	0.985	0.961–1.009	0.208
MELD ≥21	2.051	1.029–4.088	0.041^*∗*^

^*∗*^*P*value <0.05 was considered significant. AH, alcoholic hepatitis; HB, haemoglobin; PLT, platelet count; ALB, albumin; PT-INR, prothrombin time-International Standardization Ratio; MELD score, model for end-stage liver diseases score.

## Data Availability

The data that support the findings of this study are available from the corresponding author upon reasonable request.
